# Clinical team debriefing post-critical events: perceptions, benefits, and barriers among learners

**DOI:** 10.3389/fmed.2024.1406988

**Published:** 2024-11-20

**Authors:** Michelle Imperio, Kristin Ireland, Yiqing Xu, Rania Esteitie, Laren D. Tan, Abdullah Alismail

**Affiliations:** ^1^Department of Medicine, School of Medicine, Loma Linda University Health, Loma Linda, CA, United States; ^2^Division of Pulmonary, Critical Care, Hyperbaric and Sleep Medicine, Department of Internal Medicine, School of Medicine, Loma Linda University, Loma Linda, CA, United States; ^3^Department of Cardiopulmonary Sciences, School of Allied Health Professions, Loma Linda University Health, Loma Linda, CA, United States; ^4^Department of Pulmonary and Critical Care Medicine, Central Michigan University, Covenant HealthCare, Saginaw, MI, United States

**Keywords:** team debrief, medical education, post critical event, cardiopulmonary arrest, cognitive load, emotions, anonymous survey

## Abstract

**Background:**

Clinical team debriefings (TD) following critical events are pivotal in promoting team learning and enhancing patient outcomes. Despite their importance, perceptions and practices surrounding these debriefings remain under-researched. The purpose of this study was to explore learners’ perceptions and experiences regarding debriefing practices, investigate correlations or discrepancies within those perceptions and experiences, and identify recommendations and potential practice improvements for clinical educators.

**Methods:**

This was a cross-sectional anonymous survey of healthcare professionals, including medical students, medical residents, nursing students, and respiratory therapy students. The survey was sent to respiratory therapy programs, nursing programs, internal and emergency medicine and pediatric residency programs in southern California and Michigan. The variables surveyed included demographics, team debriefing experience, code experience, TD perceptions, emotional status, cognitive load, and the benefits and barriers of conducting post-code TD. Emotional status and cognitive load were assessed using validated surveys by Paas et al. and Barrett and Russell.

**Results:**

Of the 184 participants, 56% (*n* = 104) were female. The mean cognitive load was 6.14 ± 1.6. A notable negative correlation was found between mental effort in recent real code experiences and emotional scales: “tense: calm” (*r* = −0.210; *p* = 0.018), “nervous: relaxed” (*r* = −0.234; *p* = 0.008), and “stressed: serene” (*r* = −0.258; *p* = 0.004). While 68.5% had attended a cardiopulmonary arrest event, only 34.9 had TD after their most recent code, and only 48.4% reported ever having a post-code TD. Notably, nurses (75.4%) and attending physicians (73.8%) predominated these debriefings. Debriefings averaged 9.30 min (SD = 7.30) with a median of 6 min. The most recognized benefits were identifying areas of systems/process improvement and promoting teamwork and solidarity within the code team participants. The most commonly recognized barriers were lack of time and wanting a more senior person to initiate TD.

**Conclusion:**

The results of this study show a relatively low TD occurrence despite the high value learners attribute to TD. Addressing this inconsistency requires structured approaches, dedicated time, and an understanding of barriers. Recognizing the significant cognitive and emotional loads on learners further accentuates the need for structured post-event debriefings. Addressing these challenges with multi-disciplinary participation can enhance debriefing outcomes.

## Introduction

Healthcare workers in critical event responses, such as those often encountered in the intensive care unit (ICU), emergency department (ED), and acute medical floors, are routinely exposed to potentially traumatic situations. These events may include a rapid decline in patient status requiring immediate attention and potentially critical care interventions (“rapid response”), cardiorespiratory failure (“code”), and patient death. Participation in such events puts healthcare workers at risk of psychological trauma and even post-traumatic stress disorder; this risk appears to be higher in persons with less experience, such as intern physicians (1, 2).

Debriefing after such events allows participants and onlookers to process the strong emotions arising from the event and accompanying stress response and identify possible areas of process improvement for similar future situations (3, 4). Healthcare workers, when surveyed, acknowledge the benefits and usefulness of a debrief session (2, 3, 5–8), and the importance of a post-code debrief session has been emphasized in practice guidelines such as the American Heart Association CPR guidelines and the International Liaison Committee on Resuscitation guidelines (9, 10). In addition, team debrief (TD) has been reported to improve psychological safety for learners by empowering them to contribute to the discussion, clarifying expectations, and fostering inclusiveness (11). Various investigations have also been done regarding optimal techniques for conducting a post-critical event debrief session. However, much remains yet to be determined regarding best practices and specifics of how a debrief is conducted when adapted to the local situation (5, 12–14). Despite the recognized benefits of the post-code team debrief, as few as 1 in 7 hospitals frequently conduct a debriefing session immediately after in-hospital cardiac arrest events (10). In one quality improvement project, up to 70% of subjects reported never having participated in a post-critical event debrief (15). In another study, 50% of subjects reported little to no debriefing experience, and only 15% reported frequently experiencing a debriefing session after a critical event (16). Various barriers to the regular implementation of a debriefing session have been identified, including time constraints, workload, lack of a trained facilitator, debrief not initiated by a more senior participant, lack of administrative support, and fear or discomfort, among others (3, 6). Among students, debriefing has been demonstrated to be a valuable educational tool to encourage reflection, promote self-awareness of skills, and promote transfer of learning (17, 18). The purpose of this study was to examine debriefing practices and perceptions among learners, with a particular interest in correlations or dissimilarities between these perceptions and experiences. We hypothesized that learners value TD, and we sought to identify recommendations and potential improvements for clinical educators based on these findings.

## Methods

This study was approved by the Institutional Review Board of Loma Linda University Health as an exempted study. A cross-sectional anonymous questionnaire was designed and internally validated by a physician, respiratory therapist, and statistician. Inclusion criteria were medical trainees from the following professions: medical residents training in internal medicine, pediatrics, combined internal medicine-pediatrics, combined internal medicine-anesthesia, or emergency medicine; nursing students; respiratory therapy students; and medical students. Subjects were excluded if they were nonresidents/students or if they did not provide consent to participate in the study.

### Subject recruitment

The survey was emailed to program directors of selected nursing school, medical school, and medical residency programs in southern California and Michigan to be sent out to their students and residents. It was emailed to all respiratory therapy program directors in the United States. Selection of programs was based on availability and access. For respiratory therapy, program directors’ emails are publicly available on the respiratory therapy accreditation website, so we were able to send the survey to them all. For the nursing and medicine programs, investigators sent the survey to local programs in their respective geographical areas based on convenience and availability of access. Snowball sampling was also used among participants to increase awareness about the study. The process for snowball sampling was not directly controlled; rather, subjects and their faculty were encouraged to share the survey with their colleagues, potentially widening the recruited sample. To protect anonymity, we limited collection of data that could identify specific programs. Responses were collected between July 2021 and September 2022.

### Survey questions

Consent was embedded within the survey, with the questions only made available to participants who consented to participate. Responses were anonymous. Survey questions included demographics, TD experience, code experience, perceptions about TD, emotional status, cognitive load, and benefits and barriers to conducting a post-code TD.

Emotional status was assessed using a validated instrument by Feldman Barrett and Russell (19). This instrument consists of eight bipolar descriptors: tense/calm, nervous/relaxed, stressed/serene, upset/contented, sad/happy, depressed/elated, lethargic/excited, and bored/alert. Participants were asked to provide ratings on an eight-point Likert scale, with values ranging from −2 to +2 in 0.5 increments. Similarly, the measure of cognitive load drew upon the established scale by Paas et al. (20). Here, participants were instructed to denote their perceived mental effort on a 9-point Likert scale, where a score of 1 signified “very, very low mental effort” and a score of 9 represented “very, very high mental effort.” These scales have been combined in previous studies to assess participants’ emotional and cognitive load (21–23).

### Data analysis

Data were analyzed using SPSS version 28. Initial analyses involved summarizing data using frequency distribution and percentages to understand the dataset comprehensively. Correlation analysis was specifically employed to determine the relationship between emotional status and cognitive load. Descriptive statistics were calculated for continuous variables, including means, standard deviations, and ranges. Frequencies and percentages were ascertained for categorical variables. The level of significance was set at *p* ≤ 0.05.

## Results

186 participants responded to the study, and 184 agreed to participate. 56% (*n* = 104) were female. Among the 83 medical residents, 31.3% (*n* = 26) were interns (post-graduate year 1), 33.7% (*n* = 28) were in their post-graduate year two (PGY2), 30.1% (*n* = 25) were in their post-graduate year three (PGY3), 3.6% (*n* = 3) were in their post-graduate year four (PGY4), and 1.2% (*n* = 1) was in their post-graduate year five (PGY5) or above. The majority were in internal medicine with 55.4% (*n* = 46), followed by 25% (*n* = 21) in emergency medicine. Among the student population (*n* = 101), 55.4% (*n* = 56) were respiratory students, 35.6% (*n* = 36) were medical students, and 8.9% (*n* = 9) were nursing students. Most students were in their first year (34.7%, *n* = 35) and second year (37%, *n* = 36.6) of training (respiratory therapy = 2-year program); (Table 1).

**Table 1:** Subjects demographics and characteristics.

		*n*	%
Gender			
	Male	80	43.5
	Female	104	56.5
Profession			
	Medical student	36	19.6
	Nursing student	9	4.9
	Respiratory student	56	30.4
	Medical resident	83	45.1
Year of training in residency			
	Intern/PGY-1	26	31.3
	PGY-2	28	33.7
	PGY-3	25	30.1
	PGY-4	3	3.6
	PGY-5 or above	1	1.2
Type of residency programs			
	Internal medicine	46	55.4
	Emergency medicine	21	25.3
	Pediatrics	3	3.6
	Med-peds (internal medicine/pediatrics combined program)	3	3.6
	Med-anesthesia (internal medicine/anesthesia combined program)	4	4.8
	Prelim (e.g., intern year training prior to beginning further training in other medical specialties such as radiology, anesthesia, ophthalmology, dermatology, etc.)	6	7.2
Program year (MD, RT, RN students)			
	1^st^ year	35	34.7
	2^nd^ year	37	36.6
	3^rd^ year	18	17.8
	4^th^ year	10	9.9
	5^th^ year or higher	1	1.0
Type of acute care training certifications			
	Basic Life Support (*n* of 184)	166	90.2
	Advanced Cardiac Life Support (*n* of 184)	109	59.2
	Pediatric Advanced Life Support (*n* of 184)	34	18.5
	Neonatal Resuscitation Program (*n* of 184)	21	11.4
Real code experience			
	Yes	126	68.5
	no	58	31.5
Type of code attended			
	Adult	123	66.8
	Pediatric	17	9.2
	Neonatal	11	6.0

### Debrief experience

More than half of the participants reported attending a cardiopulmonary arrest event (68.5%, *n* = 126), and 34.9% (*n* = 44) reported a TD in their most recent real code experience. 51.6% (*n* = 65) had never experienced any post-code TD. Of those who had attended real codes, 84.1% (*n* = 106) had only attended an adult code. Only 4.8% (*n* = 6) of participants had attended all code types (adult, pediatric, and neonatal).

During their most recent TD, 33/61 (54.1%) responded that the attending physician initiated the debriefing. Nurses were the professionals most often present for TD (75.4%, *n* = 46), followed closely by the attending physician (73.8%, *n* = 45), senior resident (70.5%, *n* = 43), and respiratory therapist (63.9%, *n* = 39). When asked about the topics addressed in their most recent TD, 49/61 (80.3%) reported “access for possible process/systems improvement,” 39/61 (63.9%) reported “emotional processing,” 32/61 (52.5%) reported “constructive criticism for individual performance,” and 24/61 (39.3%) reported “moment of silence or reverence for the patient.” The mean duration for the most recent TD was 9.30 min (SD = 7.30), and the median duration was 6 min.

### Perceptions on how TD should be conducted

**Figure 1:**
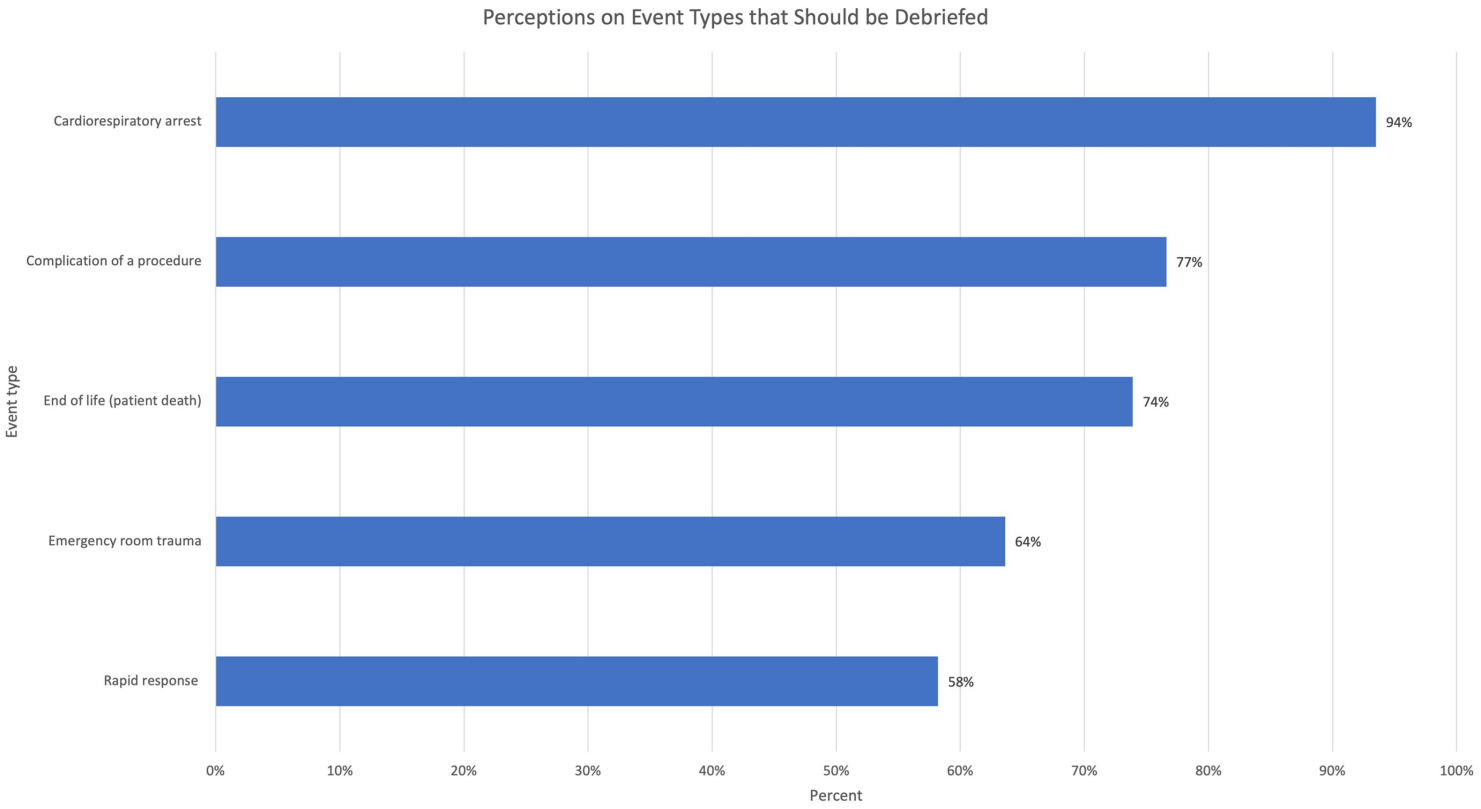
Percentage of respondents who indicated a need for post-event team debrief (TD) for various critical event types.

**Figure 2:**
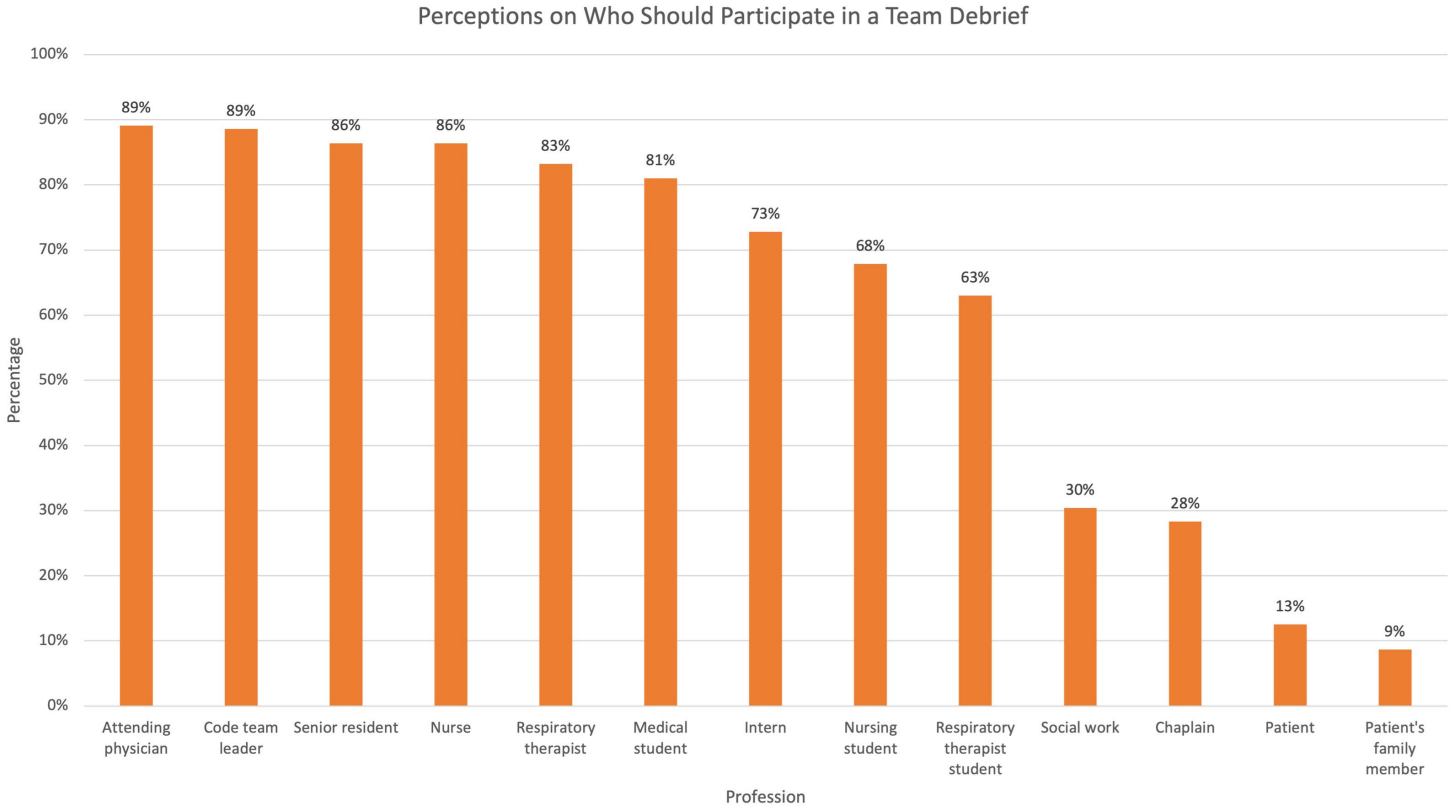
Percentages of respondents endorsing team debrief participation by various professions and roles.

**Figure 3:**
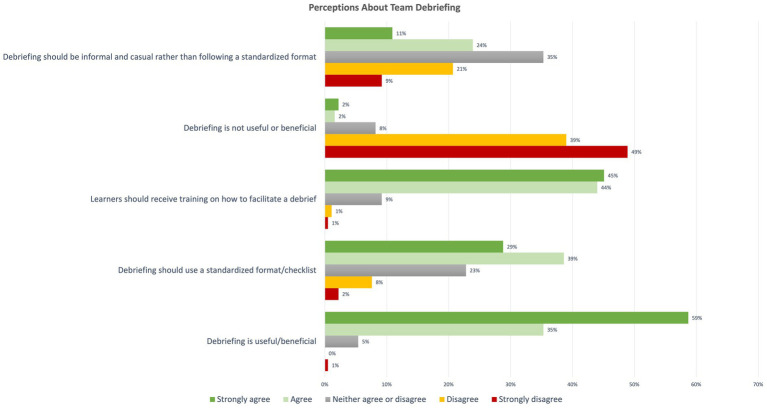
Distribution of responses indicating level of agreement with various statements regarding team debrief.

When asked about their perceptions on TD, 45.1% (*n* = 83) reported that the attending physician should initiate TD, and 44.6% (*n* = 82) reported that the code team leader should initiate TD. Most respondents believed cardiorespiratory arrest events should be debriefed (94%, *n* = 172), and many responded that other types of critical events should also be debriefed (Figure 1). 74% (*n* = 136) of the participants reported that TD should occur immediately after the critical event. Survey participants generally believed all medical staff members including physicians, residents, nurses, and respiratory therapists should participate in TD, with fewer responses stating students or other disciplines such as chaplain or social work should attend, and even fewer who believed the patient or family should attend (Figure 2). The majority believed that debrief is beneficial and useful, learners should receive debrief training, and TD should be standardized using a checklist (Figure 3).

### Benefits and barriers of TD

**Figure 4:**
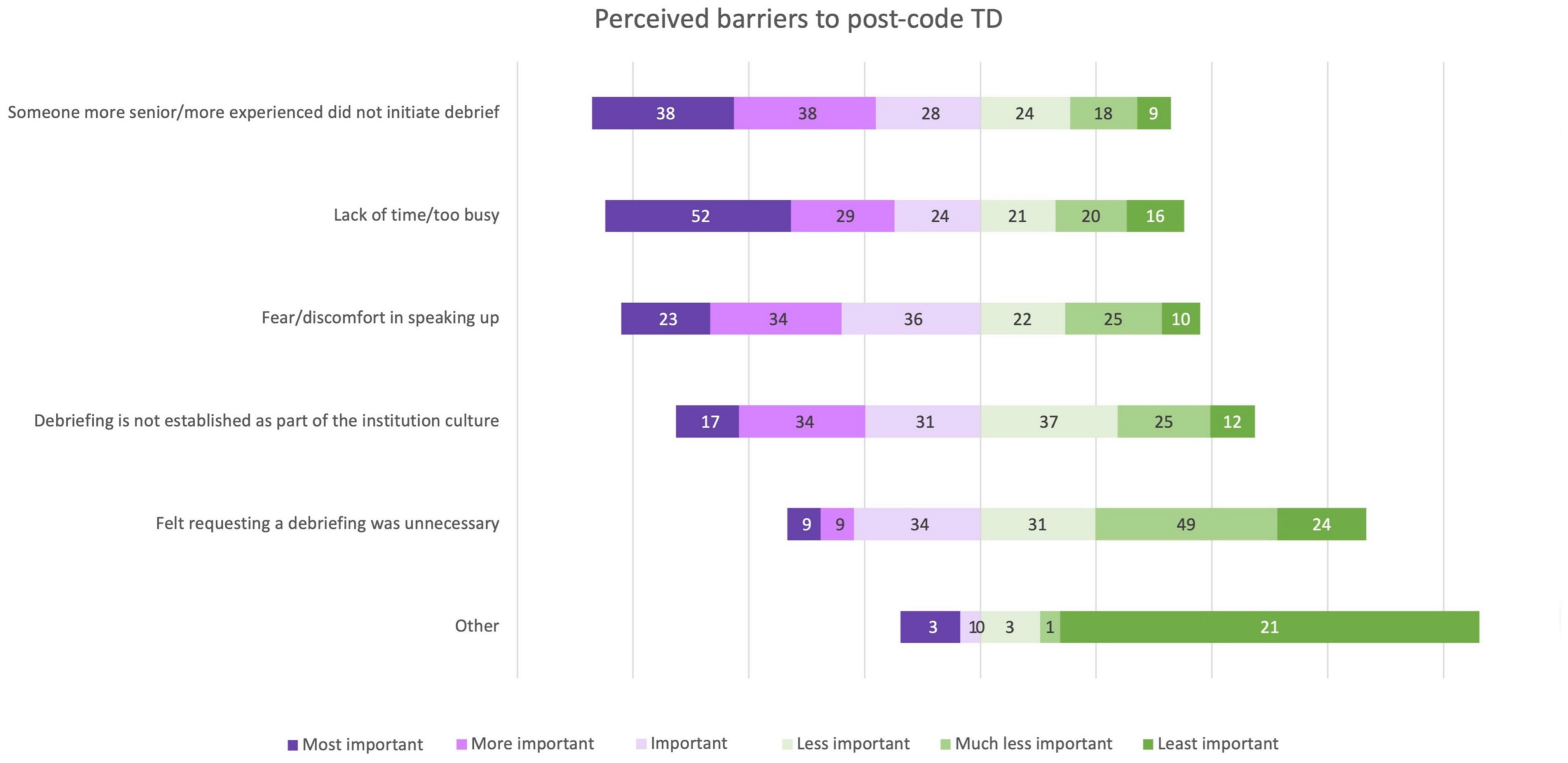
Distribution of respondents’ perceptions ranking significance of various potential barriers to post-code team debrief.

**Figure 5:**
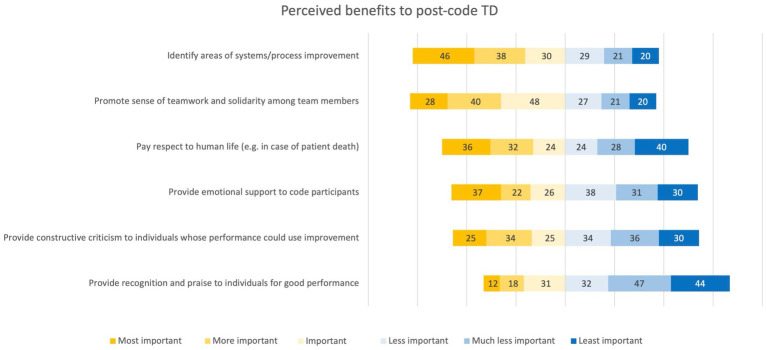
Distribution of respondents’ perceptions ranking significance of potential benefits of post-code team debrief.

Identifying areas of process improvement and promoting a sense of teamwork and solidarity were the most highly ranked benefits of TD. The most commonly reported barriers were wanting someone more senior or more experienced to initiate the debrief and a lack of time (Figures 4, 5). Table 2 further shows respondents’ ranking of most to least important benefits and barriers of team debrief on a 6-point Likert scale. Providing recognition and praise to team members for good performance was ranked as the least important benefit to TD, and feeling that TD was unnecessary was ranked as the least likely barrier to TD.

**Table 2:** Benefits and barriers of team debrief (reported in median interquartile range, mean and standard deviation).

Statement		Median IQR
		Mean ± SD
Benefits		
Identify areas of systems/process improvement		3.00(1.25–4.00) 3.01 ± 1.678
Provide emotional support to code participants		4.00(2.00–5.00) 3.51 ± 1.740
Pay respect to human life (e.g., in case of patient death)		3.50(2.00–5.00) 3.52 ± 1.843
Provide recognition and praise to individuals for good performance		4.00(3.00–5.00) 4.17 ± 1.530
Provide constructive criticism to individuals whose performance could use improvement		4.00(2.00–5.00) 3.61 ± 1.676
Promote sense of teamwork and solidarity among team members		3.00(2.00–4.00) 3.18 ± 1.545
Barriers		
Lack of time/too busy		2.50(1.00–4.00) 2.85 ± 1.724
Someone more senior/more experienced did not initiate debrief		3.00(2.00–4.00) 2.83 ± 1.534
Debriefing is not established as part of the institution culture.		3.00(2.00–4.00) 3.35 ± 1.454
Fear/discomfort in speaking up		3.00(2.00–4.00) 3.15 ± 1.499
Feeling that requesting a debriefing was unnecessary		4.00(3.00–5.00) 4.12 ± 1.377

1 = most important, 6 = least important.

### Cognitive load and emotions

The mean cognitive load reported by respondents for their most recent code was 6.14 ± 1.6 (6 = “rather high mental effort”; 7 = “high mental effort). Respondents reported being more alert than bored (scale −2.00 to +2.00, mean 1.5, SD 0.8), more nervous than relaxed (mean − 0.5, SD 1.1), more stressed than serene (mean − 0.46, SD 1.0), and more excited than lethargic (mean 0.45, SD 0.95). There was no significant difference in reported emotions or cognitive load between those with and without TD in their most recent code.

### Correlation analysis of mental effort and emotions

There was a significant negative correlation between the amount of mental effort invested in the most recent real code experience and emotional scales: “tense: calm” (*r* − 0.210; *p* = 0.018), “nervous: relaxed” (*r* = −0.234; *p* = 0.008), and “stressed: serene” (*r* = −0.258; *p* = 0.004) (Table 3). Participants reporting higher levels of mental effort were likely to rate their emotional level as more tense, nervous, and stressed during their recent code experience.

**Table 3:** Median and Interquartile ranges and mean and standard deviations for emotions.

Emotions	Median (IQR)	Correlation to cognitive load (*r*)	*p*
	Mean ± SD		
Tense: Calm	−0.500(−1.500–1.000) −0.329 ± 1.262	−0.210*	0.018*
Nervous: Relaxed	−0.750(−1.500–0.500) −0.500 ± 1.101	−0.234*	0.008*
Stressed: Serene	−0.500(−1.125–0.500) −0.464 ± 1.042	−0.258*	0.004*
Upset: Contented	0.500(−0.500–0.625) 0.091 ± 1.100	−0.157	0.079
Sad: Happy	−0.500(−1.000–0.500) −0.357 ± 0.890	−0.012	0.896
Depressed: Elated	−0.500(−0.500–0.500) −0.052 ± 0.921	0.115	0.200
Lethargic: Excited	0.500(−0.500–1.000) 0.452 ± 0.958	0.086	0.337
Bored: Alert	2.000(1.000–2.000) 1.504 ± 0.814	0.086	0.336

*p-value < 0.05.

In addition, correlation analysis between each emotional statement with cognitive load/mental effort.

## Discussion

This project was initiated based on the hypothesis that learners find TD worthwhile and effective. We sought to explore participants’ experiences and perceptions surrounding post-code TD, particularly focusing on correlations or dissimilarities between perceived and experienced reality.

Our study findings are consistent with previous research demonstrating a low reported rate of TD occurrence despite a strong preference for post-code TD (16, 24). Participants also valued TD training but only rarely reported receiving such training. We anticipated this discrepancy between desired and actual TD occurrence rates and speculated on possible reasons for barriers to post-code TD.

### Perceptions vs. reality: “TD takes too long”

In our study, learners reported a lack of time as one of the most common barriers to post-code TD. However, this barrier may be based on the misconception that TD requires a long time to complete, when in reality over three-quarters of reported TD lasted only 10 min or less, and only two survey participants with TD (3%) reported a duration of 30 min or more (one 30 min, one 45 min). We did not collect data regarding the amount of time spent on active resuscitation in survey participants’ most recent code, but we speculate that the few cases of TD that took the longest may be associated with a longer duration of the preceding code event, involving a larger team and more additional interventions. These “mega-codes” presumably require more time to debrief, and future studies should investigate further the association between code duration and the following TD session.

Regardless, our findings highlight the need for clinician educators to reframe trainees’ and bedside clinicians’ perception of time spent on TD, emphasizing TD’s importance as an integral part of the code process as well as the low time investment required in most cases (25, 26). As these precious minutes may seem substantial to the busy clinician, support from an organizational level is needed to reinforce the importance of TD within the institution culture. Furthermore, as trainees learn from observing their preceptors, bedside clinicians should model an attitude that prioritizes post-code TD as an essential element of resuscitation management. Only then can we begin to undermine the misconception that “TD takes too long.”

### Perceptions vs. reality: “The code team leader should always initiate TD”

Hierarchy appears to be another significant barrier to post-code TD, as survey participants frequently felt that someone more senior should initiate debriefing. This may be an expectation brought on by personal experience. Of those with TD following their last code, the attending physician was most frequently cited as having initiated the TD, and most survey participants believed that the attending physician or code team leader should initiate TD. However, a significant minority of TD were initiated by other team members, including nurses, respiratory therapists, and medical trainees.

Even without a strong hierarchical culture or perceived power differential among code team members of varying levels of experience, a lack of knowledge or confidence in facilitating a TD likely also contributes to learners’ hesitance to initiate TD. Most survey participants agreed or strongly agreed that learners should receive training on how to facilitate TD, and one participant commented that simulation training for TD would be helpful. However, nearly two-thirds of survey respondents had received no such training.

These results suggest that for TD to become standard practice, efforts should ensure TD training for all code team participants and emphasize code team leaders’ role in prioritizing and initiating post-code TD. Another possible solution is empowering learners to request a debrief, facilitating TD even when learners feel unable to initiate TD themselves due to inadequate TD training or a perceived hierarchy barrier (27–29).

### Perceptions vs. reality: “There is insufficient psychological safety for TD”

Fear or discomfort in speaking up, while less frequently cited as the most significant barrier to TD, is not inconsequential. Part of this reluctance may involve a lack of emotional neutrality, including the desire to avoid discussing possible medical errors. Creating an atmosphere where people feel safe discussing potentially uncomfortable topics is not always possible but is essential to facilitating effective TD (8, 30). The importance of such an atmosphere, termed “psychological safety,” for effective TD has been previously described (11). Although a thorough discussion of the multitude of factors that interact to promote psychological safety is outside the scope of our study, one possible small step forward is to empower learners to speak up and request TD (11). Ideally, such change implementation should take a “top-down” approach, starting with educators and leaders to normalize requesting TD, thus creating a precedent for learners to follow. Learners typically occupy a low-status, low-power role within the clinical hierarchy, and the simple act of validating the importance of their requests, placing them on equal footing with staff and the rest of the code team, can speak volumes in setting the tone for subsequent discussion (8, 29, 30).

Once psychological safety is established, all team members, not only senior staff but also early career individuals and learners, can be empowered to request, facilitate, and participate in TD. This has been shown to improve communication, reduce medical errors, and promote mental health (11, 31). Such training should be incorporated into healthcare education programs for both faculty and learners.

### Other findings

We speculated that mental effort would be perceived to be lower in cardiorespiratory arrest events with a TD compared to those without a post-code TD and that emotions would be more positive with a post-code TD; however, we did not find a significant difference between those with vs. without post-code TD. This may be due to a small sample size or to other factors not captured by the results of our study, such as duration or clinical complexity of cardiopulmonary resuscitation efforts. Future investigations should consider these factors.

Of note, the average cognitive load reported by study participants was 6.1 on the provided mental effort scale, corresponding to a “rather high mental effort” invested in the code event. A certain amount of “mental stress” is essential to maximize learning without causing so much of a load as to hinder learning, as has been seen with cognitive loads above 7 (22). Learners in our study therefore experienced, in general, optimal cognitive load during code events. It has been reported that cognitive load and emotions play a vital role in learning [and may play a role in learner performance and intrinsic motivation] (32). While this is encouraging, our results demonstrate that despite reporting favorable learning conditions during the code itself, learners still desire TD following the event, yet TD only occasionally occurs.

Our survey was sent to healthcare profession trainees of different disciplines and medical specialties, as code events require multidisciplinary cooperation and occur in a variety of environments. We initially hoped to compare responses between members of different health professions, but as the sample sizes from members of different health professions were so varied and our overall sample size was small, an unbiased direct comparison is likely not possible.

Many different frameworks, tools, and models of carrying out TD have been described in existing literature, and we sought to explore learners’ perspectives on these factors (11, 14, 25, 26, 33). Survey participants in our study had varied preferences in the timing (immediate vs. delayed) and format (structured vs. informal) of TD and who should attend TD. Interestingly, while most learners agreed that healthcare workers, from the attending physician to even students, including medical students, RT students, and nursing students, should attend post-code TD, only a small fraction believed that the patient or the patient’s family should attend TD (12.5 and 8.7%, respectively). Although previous literature supports the value of having family members present during the code/resuscitation, we presume that survey participants’ preference to exclude family members from debriefing reflects the importance of using TD to review systems, discuss process improvement issues, and address any medical errors that may have occurred (34–38).

### Limitations

Our study is not without limitations. The nature of this cross-sectional study means there may be a recall or selection bias. We cannot exclude the possibility that residents with stronger sentiments about their TD experience, for instance, those who more keenly felt a deficiency in their TD experience frequency, were more motivated to participate in this voluntary survey. On the other hand, “preferred response” pressures may have caused participants to indicate that debriefing occurs with greater frequency than actually experienced. However, the anonymous nature of our survey was intended to decrease such factors. Also, while our questions regarding debrief experience specifically asked participants to describe their “most recent code,” answers did not distinguish experiences based on time elapsed since the event. This then raises the possibility of imperfect recall, particularly for code experiences that may have happened in the distant past. However, we designed this study as a form of event recall to help us first answer the basic question of whether learners believe the TD experience needs to be improved or expanded upon, based on their experience and recollection of the event, and we were able to address this with our findings. Finally, our findings represent learners’ viewpoints within selected professions and institutions but may not represent the experiences at other institutions. Therefore, it is difficult to generalize the findings of this study, especially with the low sample size. For example, our nursing student sample was from only one institution and the response and participation were very low, limiting our ability to generalize our findings regarding nursing students’ perspectives. The nursing profession is a critical and core profession in any code event; therefore, we strongly recommend future researchers ensure recruitment of more nursing students to obtain their perspective. Furthermore, we are unable to provide an estimated response rate due to the inability to calculate an estimated pool. Even with the wide distribution of our invitation letter to program directors to be shared with their students, we believe that data collection during the COVID-19 period may have negatively impacted our response rate.

Finally, while our results identified potential gaps in current practice regarding learners’ experiences with post-code TD, change implementation was outside the scope of our study. Future research should focus on quality improvement projects using methods such as [Plan, Do, Check, Act] to ensure sustainability. A recent community-based case study successfully implemented a clinical debrief process in the emergency room during the COVID-19 pandemic (7). Their model was successful among staff, and future research should examine the feasibility and sustainability of such a model among students. One possible approach is to incorporate TD simulation training into students’ educational programs, followed by collaboration with clinical sites to collect data and measure success (18, 39, 40). This may also help students achieve a shared mental model in critical care areas (40).

## Conclusion

Post-code debriefing has been shown to be beneficial for both process improvement and emotional support for code participants and is recommended in current cardiac arrest management guidelines. However, similar to previous findings, our study demonstrates that post-code TD occurs infrequently (10, 14, 15). Furthermore, our observational study provides valuable insight into the perspectives of learners regarding code experiences and perceptions regarding post-code TD. As most learners expect code team leaders to initiate TD, post-code TD-specific training should emphasize the importance of TD as an essential part of the code team leader role. Notwithstanding learners’ expectations, however, any code team participant may initiate a TD, which should also be emphasized in TD training. Regardless of the structure and format of post-code TD, efforts should be undertaken to establish post-code TD as part of institution culture, emphasizing the high benefits relative to time cost and focusing on establishing a culture of psychological safety for such critical events to be discussed.

## Data Availability

The raw data supporting the conclusions of this article will be made available by the authors, without undue reservation.
